# Cryopreservation of glucose-6-phosphate dehydrogenase activity inside red blood cells: developing a specimen repository in support of development and evaluation of glucose-6-phosphate dehydrogenase deficiency tests

**DOI:** 10.1186/1475-2875-12-286

**Published:** 2013-08-20

**Authors:** Maria Kahn, Nicole LaRue, Pooja Bansil, Michael Kalnoky, Sarah McGray, Gonzalo J Domingo

**Affiliations:** 1 PATH, 2201 Westlake Avenue, Suite 200, Seattle, WA 98121, USA; 2 Tsunga Analytics, 746 22nd Ave E, Seattle, WA 98112, USA

**Keywords:** *Plasmodium vivax*, Haemoglobinopathy, Glucose-6-phosphate dehydrogenase, Malaria, Diagnostic tests, Point-of-care

## Abstract

**Background:**

Glucose-6-phosphate dehydrogenase (G6PD) deficiency is a common human enzyme deficiency. It is characterized by abnormally low levels of G6PD activity. Individuals with G6PD deficiency are at risk of undergoing acute haemolysis when exposed to 8‒aminoquinoline-based drugs, such as primaquine. For this reason it is imperative to identify individuals with G6PD deficiency prior to administering these anti-malarial drugs. There is a need for the development and evaluation of point-of-care G6PD deficiency screening tests suitable for areas of the developing world where malarial treatments are frequently administered. The development and evaluation of new G6PD tests will be greatly assisted with the availability of specimen repositories.

**Methods:**

Cryopreservation of erythrocytes was evaluated as a means to preserve G6PD activity. Blood specimens from 31 patients including ten specimens with normal G6PD activity, three with intermediate activity, and 18 with deficient activity were cryopreserved for up to six months.

**Results:**

Good correlation in G6PD activity between fresh and cryopreserved specimens (R^2^ = 0.95). The cryopreserved specimens show an overall small drop in mean G6PD activity of 0.23 U/g Hb (P=0.23). Cytochemical staining showed that intracellular G6PD activity distribution within the red blood cell populations is preserved during cryopreservation. Furthermore, the mosaic composition of red blood cells in heterozygous women is also preserved for six months or more. The fluorescent spot and the BinaxNOW qualitative tests for G6PD deficiency also showed high concordance in G6PD status determination between cryopreserved specimens and fresh specimens.

**Conclusions:**

A methodology for establishing a specimen panel for evaluation of G6PD tests is described. The approach is similar to that used in several malaria research facilities for the cryopreservation of parasites in clinical specimens and axenic cultures. Specimens stored in this manner will aid both the development and evaluation of current and emerging G6PD tests. The availability of G6PD tests is a critical bottleneck to broader access to drugs that confer radical cure of *Plasmodium vivax*, a requirement for elimination of malaria.

## Background

Glucose-6-phosphate dehydrogenase (G6PD) deficiency is the most commonly found human enzyme deficiency, affecting over 400 million people worldwide [1-4]. It is characterized by abnormally low levels of G6PD activity. Individuals with diminished G6PD activity are susceptible to cellular oxidative damage and can exhibit symptoms including haemolytic anaemia and jaundice in response to a number of causes, most commonly infection or exposure to certain medications. In particular, treatment with anti-malarial drugs such as those in the 8‒aminoquinolone group (e g, primaquine, pamaquine and tafenoquine) can cause acute haemolysis in people with G6PD deficiency [4,5]. Primaquine is also currently the only drug available that is capable of radical cure of *Plasmodium vivax*. Unfortunately, the prevalence map for G6PD deficiency overlaps significantly with that for *P. vivax* prevalence, possibly due to an evolutionary advantage in people with G6PD deficiency against severe malaria morbidity [6]. Thus, people who most need these drugs have a relatively high chance of also carrying the G6PD deficiency trait, putting them at risk of adverse reactions.

As malaria programmes move towards elimination, radical cure of *P. vivax* becomes more critical, and the widespread use of 8-aminoquinolones will be essential. A major bottleneck to the adoption of these drugs is determining the G6PD status of a patient presenting with malaria. There is a need for a G6PD deficiency test that can be used in the same context of malaria rapid diagnostic tests. The gold standard for G6PD testing is a quantitative test that measures nicotinamide adenine dinucleotide phosphate production by the enzyme with a UV-spectrophotometer, and the standard of care is the fluorescent spot test, which requires a UV lamp and a water bath. Neither of these tests is really amenable to the point of care. There is one commercial test, the Alere BinaxNOW test, on a lateral flow test platform, but it is too expensive and the working temperature is too low to be practical for adoption in malaria-endemic regions [7]. There is a second test on the same platform that is in late-stage development and evaluation [8]. Development and evaluation of these tests would benefit greatly from the availability of repositories of specimens highly characterized for G6PD activity and genotype.

Cryopreservation of red blood cells has been an essential technique used in blood banks for many years. The cryopreserved red blood cells are often used in transfusions and in serological testing. Many blood group reference laboratories have the ability to freeze, reconstitute and analyse red blood cells that were collected months or years earlier [9-11]. Over the years several methods have been used to preserve the metabolic functions of red blood cells [12,13]. In this report, the feasibility of developing a G6PD specimen repository by cryopreservation is evaluated. Enzyme stability is demonstrated by quantitative and qualitative G6PD enzyme assays. G6PD activity distributions inside the red blood cells are also preserved as demonstrated by flow cytometry. The methods presented in this article are very similar to those used to cryopreserve blood-stage *Plasmodium* parasite derived from clinical specimens or axenic culture, and is, therefore, already familiar to several malaria clinical research facilities [14,15].

## Methods

### Human subjects and specimen collection

All specimens were sourced through Bioreclamation, Inc (Westbury, NY, USA) and were collected from voluntary donors after signed consent under institutional review board protocol 2010–017 IRB. Specimens arrived at PATH in 5-mL glass ethylenediaminetetraacetic acid anticoagulant venipuncture vacuum tubes on cold packs and were stored at 4°C. Specimen processing took place between two and four days after original blood draw.

### Cryopreservation and thawing protocol

#### Freezing red blood cells

A glycerol-based protocol for cryopreservation of red blood cells was used [13]. A total of 6 mL of whole blood was transferred to a 15- mL test tube and centrifuged 1,000/g for 5 min. The plasma and buffy coats were aspirated. The red blood cells were washed with 0.9% saline, centrifuged at 1,000/g for 5 min, and the supernatant aspirated. The cells were washed until the supernatant was clear. Two volumes of Glycerolyte 57 (Baxter, USA) were added to one volume of red blood cell pellet in the following way: first, 20% of Glycerolyte 57 was added dropwise to the red blood cells while gently mixing and incubated for 10 min with gentle rocking. The remaining Glycerolyte 57 (80%) was added dropwise while gently mixing and was incubated for an additional 10 min with gentle rocking to give a final 40% weight by volume glycerol concentration. The 1.5-mL aliquots of glycerolyte-treated red blood cells were transferred into cryovials stored at −80°C.

#### Thawing red blood cells

The cryovials were removed from −80°C and thawed at room temperature [13]. The red blood cells were transferred to a 5-mL test tube. They were centrifuged at 1,000/g for 2 min, and the glycerol freezing solution was aspirated. Then 0.16 mL of 12% saline for each 0.5 mL of red blood cell pellet volume was added dropwise and with gentle shaking over a period of 5 min. The red blood cells were left standing for 3 min at room temperature. Next, 0.5 mL of 0.2% dextrose/0.9% saline was added dropwise with gentle shaking per 0.5 mL of red blood cell pellet. The red blood cells were left sitting for 2 min at room temperature. This process — adding 0.5 mL of 0.2% dextrose/0.9% saline and letting the red blood cells stand undisturbed at room temperature — was repeated until the final volume of solution added to the test tube was 4 mL. The red blood cells were then centrifuged at 1,000/g for 1 min, and 0.5 mL of the supernatant was aspirated. The red blood cells were resuspended by repeatedly inverting the test tube. The amount of 0.5 mL of 0.2% dextrose/0.9% saline was slowly added dropwise, followed by gentle shaking. After the 0.2% dextrose/0.9% saline was added, the red blood cells sat for 2 min at room temperature. This process — centrifuging, removing the supernatant, resuspending the red blood cell pellet, adding a volume of 0.2% dextrose/0.9% saline equal to what was aspirated and then standing for 1 min — was repeated with the following volumes: 1, 1.5, 2, and 4 mL. Finally, the red blood cells were centrifuged at 1,000/g for 1 min, and all of the supernatant was aspirated. The red blood cells were washed with 0.9% saline until no haemolysis was observed.

### Quantitative G6PD trinity assay

All specimens were characterized for G6PD activity in duplicate using the quantitative G6PD kit from Trinity (Cat No 345-B Trinity Biotech Plc, Ireland). Normal, intermediate and deficient Trinity controls (Cat No G6888, G5029, G5888) were run using the same method each day the assay was run. The quantitative assays were run per the kit instructions. The enzyme activity was determined at 30°C using a temperature regulated spectrophotometer (UV-1800 Shimadzu) by measuring the change in rate in absorbance at 340 nm over 5 min for reaction solutions held in UV disposable cuvettes (Brand Cat No 759150). All G6PD activity rates are provided in U/gHb. Haemoglobin concentration was determined using a Hemocue (Fisher Scientific, USA, Hb 201+ Analyzer, Hemocue Inc, No 121721, Cat No 22-601-007).

### Trinity fluorescent spot test – G6PD qualitative assay

Each individual specimen and Trinity normal, intermediate and deficient controls (Cat No G6888, G5029 and G5888) were run at three different time points (at time zero, after 5 min and after 10 min) using Whatman No 1 filter paper (Cat No 1001–150) on the qualitative G6PD assay Trinity fluorescent spot test kit (Cat No 203-A Trinity Biotech Plc, Ireland). Controls were run each day that specimens were run.

### BinaxNOW card test – G6PD qualitative test

Reproducibility of G6PD activity with fresh and thawed specimens on the lateral flow test platform was evaluated using the BinaxNOW G6PD Test (Alere, USA, Cat No 780–000). The test can only be run between 18°C and 25°C. G6PD Trinity controls (normal control G6888 and deficient control G5888) were run periodically to ensure quality performance of the BinaxNOW G6PD test.

### Flow cytometry protocol

Whole blood specimens were characterized for intracellular G6PD activity by flow cytometry as described previously [16]. This method allows observation of mosaic red blood cell populations in specimens from females by looking at the activity of G6PD in individual erythrocytes. Ten microliters of 50% haemotocrit red blood cell suspension was diluted into 90 μL of 0.9% NaCl and was combined with 100 μL of sodium nitrite (0.125 M, Sigma, USA) and incubated at room temperature for 20 min. Samples were washed three times with phosphate-buffered saline (PBS) at 3,000 rpm for 3 min and resuspended in 100 μL of PBS. The red blood cells were then combined with 18 μL of glucose (0.28 M) in phosphate-buffered saline and 6 μL of Nile Blue Sulphate (0.01% Sigma, USA) and incubated at 37°C for 90 min with the lids of the Eppendorf tubes open. After the incubation, 2.5 μL of 0.4 M potassium cyanide (Sigma, USA) was added and incubated for 5 min. Five microliters of each sample were added to 100 μL of 3% hydrogen peroxide in PBS, agitated vigorously by hand, and washed two times in PBS. Specimens were analysed using a FACScaliber™ cytometer, 10,000 events, in the FL1 channel 533 +− 30 nm.

### Statistical methods

All statistical analyses were conducted in Stata 12.0 (Statacorp, College Station, TX, USA). The mean and the standard deviation (SD) of the quantitative Trinity G6PD assay were determined for all specimens at Day 0, and all specimens were thawed between Day 0 and Day 200; summary statistics were also calculated for specimens stratified by the following cryopreservation time intervals: i) specimens thawed between Day 0 and Day 30 after cryopreservation, ii) specimens thawed between Day 31 and Day 89 after cryopreservation, and iii) specimens thawed between Day 90 and Day 200 after cryopreservation. A paired *t*-test was used to test whether there was a significant difference between the mean assay results between Day 0 and the thaw date.

To determine the correlation between the quantitative Trinity G6PD assays pre- and post-cryopreservation, data from all cryopreserved specimens were plotted against data from Day 0. A linear regression line that best fit the data was plotted, and the corresponding R^2^ was calculated. Bland-Altman analyses were conducted to visually assess the agreement between the test results over time. The mean differences, standard deviation of the difference, and a 95% tolerance-bound mean dif-ference ±1.96 SD (limits of agreement) were calculated and plotted.

In order to determine the correlation between qualitative test results, pre- and post-thaw BinaxNOW and Trinity fluorescent spot test results were compared using a McNemar’s and McNemar-Bowker test, respectively.

Finally, for a subset of four specimens for which three additional test results (in addition to Day 0) were available, generalized estimating equations with an exchangeable correlation matrix and robust standard errors were used to estimate the combined effect for the difference between test results over time, adjusting for test results at Day 0.

## Results

### Specimen description

Blood specimens from 31 patients (21 males and ten females) were cryopreserved as described in Methods. Multiple aliquots of the blood specimens were stored at −80°C. The blood specimens were then thawed at multiple time points between Day 0 and Day 200 after cryopreservation. All specimens were characterized for G6PD activity with the Trinity quantitative assay as well as by flow cytometry assay as described in Methods, both prior to cryopreservation (Day 0 data) and just after thawing. By the quantitative assay, ten specimens had normal activity, three were of intermediate activity, and 18 were deficient in G6PD activity (Table 1). By flow cytometry, four of the females displayed a population of red blood cells with a high G6PD activity and a population of red blood cells with a low G6PD activity, characteristic of females who are heterozygous for G6PD. A subset of these specimens was also tested by two qualitative tests, the fluorescent spot test and the BinaxNOW test.

**Table 1:** **Characteristics of the 31 specimens used to evaluate G6PD activity within cryopreserved red blood cells [**[16]**]**

**Phenotype by activity**	**Male**	**Female**	**Total**	**Phenotype by cytochemical staining**	**Male**	**Female**	**Total**
**Normal**	6	4	10	**Normal**	6	3	9
**Intermediate**	0	3	3	**Heterozygous**	-	4	4
**Deficient**	15	3	18	**Deficient**	15	3	18
**Total**	21	10	31	**Total**	21	10	31

G6PD activity was determined by spectrophotometry with a quantitative assay. G6PD activity within red blood cells was observed by flow cytometry.

### G6PD activity in cryopreserved specimens

The mean G6PD activity for the fresh blood samples from the 31 patients used in this study was 4.10 U/gHb (Table 2). Quantitative Trinity G6PD activity levels were determined for a total of 51 cryopreserved specimens at various time intervals between Day 0 and Day 200 (Table 2). Of these, 21 were thawed and tested between Day 0 and Day 30, 20 were thawed and tested between Day 31 and Day 89, and ten were thawed and tested between Day 90 and Day 200. For the entire combined sample set, there was a small drop in mean G6PD activity between Day 0 and the thaw date (0.232 U/gHb, P= 0.233). Similar changes in mean G6PD activity were observed for specimens cryopreserved up to 30 days and between 90 and 200 days, whilst the G6PD activity for specimens cryopreserved for 31 to 89 days was not significantly different to the mean G6PD activity at Day 0 (P= 0.629).

**Table 2:** Mean and standard deviation (SD) of G6PD enzyme activity (in U/gHb) at Day 0 and at thaw date

	**Cryopreservation time intervals (days) specimens**			
	**0 to 30**	**31 to 89**	**90 to 200**	**Sum (0 to 200)**
**Number of cryopreserved specimens (Number of corresponding fresh blood specimens)**	21 (14)	20 (20)	10 (10)	**51 (31)**
**Mean**^**a **^**(SD) G6PD activity at Day 0**	4.57 (4.22)	4.40 (3.80)	4.74 (4.05)	**4.10 (3.90)**
**Mean**^**b **^**(SD) G6PD activity at thaw date**	4.04 (4.33)	4.55 (4.68)	3.91 (3.93)	**3.87 (4.41)**
**Mean difference (SD) between Day 0 &thaw date**	0.53 (0.46)	−0.15 (0.31)	0.84 (0.09)	**0.23 (1.05)**
*P *value*	<0.001	0.629	<0.001	**0.233**

Mean^a^ (SD) G6PD activity at Day 0 is the mean of the activities in fresh blood from which cryopreserved aliquots were used over a given time interval. Mean^b^ (SD) G6PD activity at thaw date is the mean of the activities in the cryopreserved specimens thawed over a given time interval.

*Paired *t* test *P* value.

**Figure 1:**
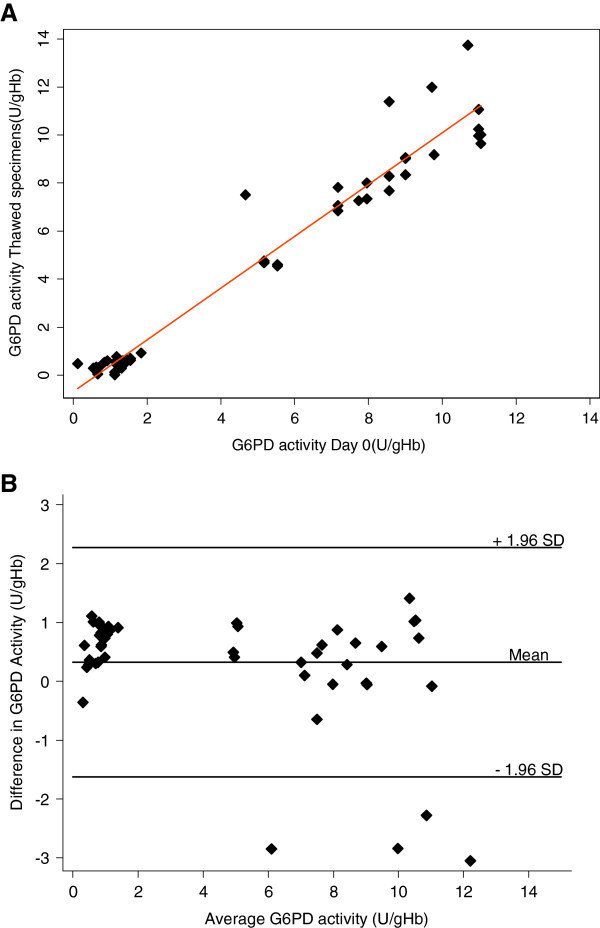
**Correlation in G6PD activity values between fresh specimens (Day 0) and cryopreserved specimens. (A)** Direct comparison of G6PD activities for fresh and cryopreserved specimens is shown. The solid line indicates the linear regression fit. **(B)** Bland-Altman plot for differences in G6PD activity between fresh and cryopreserved specimens.

A scatterplot showing the correlations of G6PD activity of specimens cryopreserved for different time intervals *vs* Day 0 (fresh specimens) are shown in Figure 1A. When compared to Day 0, all specimens, including those stratified by thaw-date interval, had strong linear correlations (P<0.001) with R^2^ values ranging between 0.9404 and 0.9953. Figure 1B shows the Bland Altman analysis, a visual comparison of test results between cryopreservation intervals and Day 0 for all specimens. For all cryopreserved specimens, the mean difference is 0.23 (95% CI: (−1.83, 2.29)), suggesting that the correlations are not biased. Similar results were observed when stratified by cryopreservation time interval. A subset of four specimens fell out of the 1.96 SD range.

**Figure 2:**
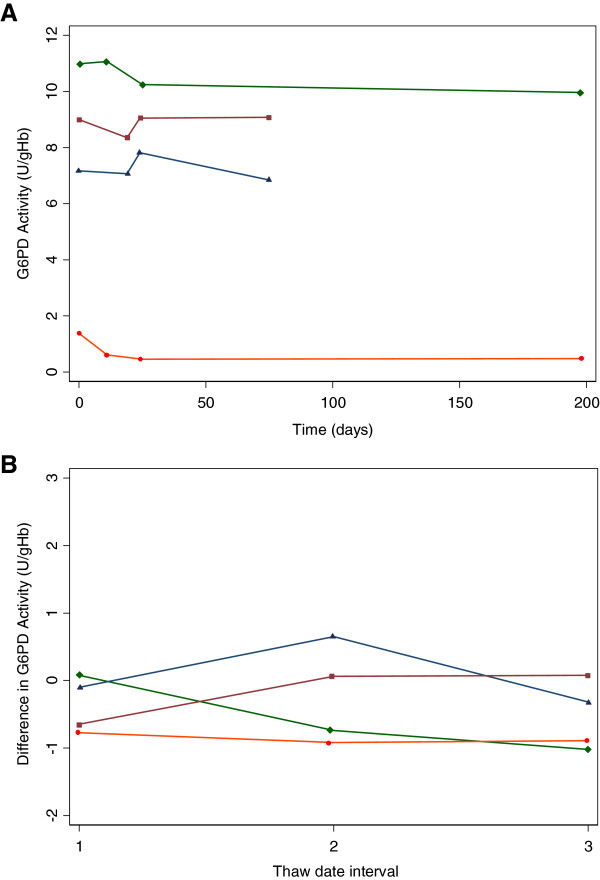
**Longitudinal G6PD activity recovered from three cryopreserved aliquots of the same specimen.** Data are shown for four specimens, three with normal G6PD activity and one with deficient G6PD activity. Specimens were cryopreserved at a single date in multiple aliquots. Aliquots of cryopreserved specimens were than thawed at multiple dates and analysed for G6PD activity. **(A)** Absolute G6PD activity at thaw dates after cryopreservation and **(B)** Difference in G6PD activity from that at Day 0. For easy comparison the dates were normalized to Days 1, 2, and 3 in Figure 2**B**.

For a subset of four specimens, three with normal activity and one deficient in G6PD activity, data were available for Day 0 plus three additional cryopreservation time intervals (Figure 2). Results from a generalized estimating equations model on repeated test results on four specimens over time indicate that time was not a significant factor in predicting the test results at any thaw date (Table 3).

**Table 3:** Parameter estimates and robust standard errors for the generalized estimating equations model of quantitative trinity results

**Parameter**	**β coefficient**	**Robust standard error**	***P *value**
Intercept	−0.50	0.350	0.153
Time (days)	−0.003	0.002	0.138
Day 0 Trinity result	1.04	0.040	0.000

### G6PD activity distribution in the red blood cell population

**Figure 3:**
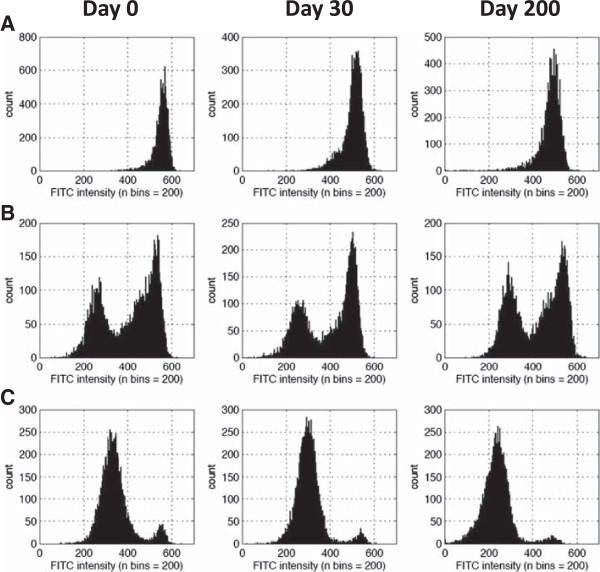
**Distribution of G6PD activity within red blood cell popoulations of fresh and cryopreserved specimens.** Intracellular G6PD activity distributions as indicated by flourscence are shown for **(A)** a normal male, **(B)** heterozygous female, and **(C)** deficient male specimens. Intracellular G6PD activity profiles are shown for Day 0 prior to cryopreservation and two separate dates at which the same specimen was thawed after cryopreservation.

Intracellular G6PD activity was observed by cytochemical staining of the cells as described previously and in the methods [16]. The distribution of G6PD activity within the red blood cell population of fresh specimens and cryopreserved specimens was observed by flow cytometry (Figure 3). Only minor changes in profiles are observed which are possibly due to the cryopreservation and thawing process (Figure 3).

### Performance of qualitative tests on cryopreserved specimens

The G6PD deficiency point-of-care test and the fluorescent spot test were performed on the stored specimens to evaluate the performance of these tests on cryopreserved specimens. Cryopreserved specimens were resuspended to a 50% haematocrit, and the tests were performed as per the product insert. Among the 21 specimens for which pre- and post-cryopreservation BinaxNOW data were available, there was 100% agreement in the test results (Table 4). For the Trinity fluorescent spot test, one specimen with normal activity by the spot test prior to cryopreservation but a low G6PD activity by the quantitative test (4.67 U/g Hb) was classified as intermediate after preservation. There were five specimens which were classified as having intermediate activities by the fluorescent spot test prior to cryopreservation and then classified as deficient after cryopreservation. These had all been classified as deficient by the quantitative test both on fresh specimens and post cryopreservation (G6PD activities of 1.27, 1.13, 1.20, 1.45, 0.12 U/g Hb). The reasons for these discrepancies are being investigated by further molecular studies. Although the agreement between the pre- and post-cryopreservation Trinity fluorescent spot test was 71.4%, the McNemar-Bowker symmetry test results indicate there was no significant difference between the test results at the two time periods (P= 0.0498) (Table 4).

**Table 4:** G6PD status after cryopreservation as determined by two qualitative tests compared to status pre-cryopreservation

**BinaxNOW G6PD deficiency test**		**Post-cryopreservation results**			
		**Normal**	**Deficient**	**Total**	
**Fresh specimen results**	**Normal**	8	0	8	
	**Deficient**	0	13	13	
	**Total**	8	13	21	
**Fluorescent spot test**		**Post-cryopreservation results**			
		**Normal**	**Intermediate**	**Deficient**	**Total**
**Fresh specimen results**	**Normal**	7	1	0	8
	**Intermediate**	0	0	5*	5
	**Deficient**	0	0	8	8
	**Total**	7	1	13	21

* The five specimens initially characterized as intermediate by the fluorescent spot test were G6PD deficient by the quantitative test.

## Discussion

The availability of clinically relevant specimens is critical in diagnostic test development and evaluation. In the case of tests for G6PD deficiency, a particular challenge is access to specimens with intermediate and low G6PD activity as well as heterozygous females. These are typically in low prevalence in the population and require significant screening to identify patients with these G6PD activity characteristics. Unfortunately, when these specimens are obtained they must be tested within a short time frame (seven to 14 days) [17]. Establishing a specimen bank enriched for specimens with intermediate G6PD activity, with deficient G6PD activity, and from confirmed heterozygous females would be of great value to both the development of tests for G6PD deficiency and to their early evaluation.

Red blood cells are routinely cryopreserved for blood-banking purposes [10,11]. The process for this results in storage of large blood volumes far beyond what is needed for G6PD testing. Additionally, the processes use automated, functionally closed systems for the glycerolization and deglycerolization steps, which are typically neither available nor appropriate outside of a blood-bank setting. Cryopreservation of red blood cells, as described in this article, can be used to establish a specimen repository at appropriate specimen volumes to support the development and evaluation of G6PD tests. Good quantitative correlation of G6PD activity in specimens that have been stored for up to 200 days (R^2^ = 0.95) is demonstrated. The specimens experience a small but significant drop in G6PD activity: 0.23 U/g Hb difference in mean activities for all specimens. A subset of these specimens experienced a more than two standard deviations drop in G6PD activity after going through the cryopreservation and thaw process (Figure 1B), this may reflect the user dependence of the process, and as the protocols have been optimized the frequency of these observations have reduced. The ability to examine G6PD activity within the red blood cells through cytochemical staining is a useful means to identify heterozygous female specimens [16,18-20]. Intracellular G6PD activity distribution in the red blood cell population was also cryopreserved remarkably well and would allow differentiation between specimens originating from a normal hemizygous male, a heterozygous female, and a deficient hemizygous male. While the data presented here demonstrates that red blood cells in EDTA can be cryopreserved with significant consistency, cryopreservation specifically for cytochemical staining may be improved by using blood specimens collected in acid citrate dextrose (ACD). Qualitative tests also showed similar performance pre- and post-cryopreservation with discordance only occurring with the fluorescent spot test for G6PD-deficient specimens, which had been misclassified originally as specimens with intermediate G6PD activity by the same test.

While the feasibility of using cryopreservation to establish specimen banks for G6PD testing has been demonstrated, it is important to recognize that the methodology is fairly user-dependent, complex and can definitely benefit from small-scale, semi-automated processes to standardize the freezing and thawing process. These processes continue to be optimized. Other approaches such as lyophilization should be explored further, especially if such approaches can reduce reliance on a cold chain infrastructure [12]. Additionally there are new technologies emerging that may provide alternative specimen storage approaches [21].

The only tools available to the malaria community to achieve radical cure of *P. vivax* and to reduce gametocyte transmission are the 8-aminoquinoline-based drugs. With the resurgence of drug resistance and a focus toward malaria elimination, there is a greater and more urgent need to make these drugs more widely available. This can only happen with broader access to G6PD testing. Current tests for G6PD deficiency do not meet this demand. New G6PD tests are required, and cost-efficient ways to support their development and evaluation will be critical to ensure good quality products enter the market.

## Competing interests

The authors declare that they have no competing interests.

## Authors’ contributions

MK and NL performed all experimental work described in this manuscript. PB and MK performed data analysis, SM contributed to the set up of the study, and GJD contributed to the study design, experimental design and the coordination and write up of the manuscript. All authors contributed to writing of the manuscript. All authors read and approved the final manuscript.
